# Effect of Nanosized NbC Precipitates on Hydrogen Diffusion in X80 Pipeline Steel

**DOI:** 10.3390/ma10070721

**Published:** 2017-06-28

**Authors:** Qiaoqi Cui, Junsheng Wu, Donghan Xie, Xiaoguang Wu, Yunhua Huang, Xiaogang Li

**Affiliations:** 1 Corrosion and Protection Center, University of Science and Technology Beijing, Beijing 100083, China; jump_summer@163.com (Q.C.); wujs@ustb.edu.cn (J.W.); xiedonghan@163.com (D.X.); guang1211@126.com (X.W.); lixiaogang99@263.net (X.L.); 2 Ningbo Institute of Material Technology & Engineering, Chinese Academy of Sciences, Ningbo 315201, China

**Keywords:** NbC precipitate, hydrogen permeation, apparent diffusion coefficient, hydrogen traps

## Abstract

In this paper, the effects of dispersed 3~10 nm NbC precipitates on hydrogen diffusion in X80 pipeline steel were investigated by means of high resolution transmission electron microscopy (HRTEM), electrochemical hydrogen permeation, and thermal desorption spectroscopy (TDS). The relationship between hydrogen diffusion and temperature was determined for Nb-free X80 and 0.055 wt% Nb X80 steel. The temperature dividing reversible and irreversible traps was measured, and the quantity of hydrogen captured by different traps was calculated. Three types of hydrogen trap were designed and applied in the test, and the results revealed that irreversible hydrogen traps formed by nanosized and coherent NbC precipitates markedly hindered hydrogen diffusion, and prolonged breakthrough time in Nb-bearing X80 steel.

## 1. Introduction

Stress corrosion cracking (SCC) and hydrogen-induced cracking (HIC) are related to the process of diffusion and enrichment of hydrogen in steel [1,2,3,4]. The diffusion coefficient of hydrogen in a material is an important parameter in the study of hydrogen diffusion behavior and the mechanism of hydrogen embrittlement. Since the first time this method was used to study hydrogen diffusion [5,6], electrochemical permeation has been frequently employed in testing due to its simplicity, high sensitivity, and flexibility. It has now become a routine method for studying hydrogen corrosion. In general, metals and alloys possess some precipitated phases and various kinds of crystal defects, such as vacancies, dislocations, grain boundaries, and phase boundaries, where hydrogen atoms can be captured. The electrochemical permeation method can study hydrogen embrittlement by measuring the apparent hydrogen diffusion coefficient (D_app_), surface hydrogen concentration (C_0_), and other parameters.

Over the years, more than 1500 studies in the literature have reported on the behavior and effects of hydrogen in steels and their welds [7]. The effects of nano V/Ti/Nb carbide or nitride precipitates, which can capture hydrogen atoms as irreversible traps, with respect to hydrogen embrittlement, have been investigated [8,9,10]. The trapping theory suggests that highly-dispersed hydrogen traps can be expected to have the strongest capture effect [11]. Many factors, such as the size and type of precipitate, can affect the values of the binding energies. It was reported that the amount of dissolved hydrogen is mainly determined by the presence of deep traps, formed by dispersed precipitates, such as NbN, NbC, and TiN, which possess different binding energies (from about 40 to 120 kJ/mol H) [12,13,14]. Meanwhile, the interfacial structure formed by the precipitate and matrix markedly affects hydrogen trapping [15]. Irreversible hydrogen trapping efficiency depends on the amount, size, and dispersity of precipitates, which result in different data and relational expressions of hydrogen diffusion [16,17,18]. Thus, the effects of nanosized NbC precipitates, as irreversible traps, on hydrogen diffusion and enrichment in X80 steel, have been studied continuously and in great detail.

In this work, optical microscope and HRTEM were employed to observe the microstructure. Effects of 3~10 nm NbC precipitates on hydrogen diffusion were studied by electrochemical hydrogen permeation method. The relationships between the D_app_ and the temperature were deduced for X80 steel, with and without NbC precipitates. Moreover, the critical temperature dividing reversible traps and irreversible traps was determined by TDS method, and the quantity of hydrogen captured by the two types of traps was calculated. At the end of this paper, the hindering effects of different hydrogen traps are revealed.

## 2. Test Materials and Methods

### 2.1. Test Materials

Two kinds of X80 steels were used in this experiment. Except for Nb, both test steels possessed the same chemical compositions, as shown in Table 1. Both steels were hot rolled at 1200 °C, and then air cooled to 830 °C for a second rolling. Immediately after rolling, test steels were water-quenched to 430~470 °C, and then cooled in air to room temperature.

### 2.2. Test Methods

#### 2.2.1. Microstructure Observation

The microstructures of both steels were obtained using an optical microscope. HRTEM was used to observe the distribution and size of precipitates, the interface of precipitate/matrix, and also to index the precipitates.

#### 2.2.2. Thermal Desorption Spectroscopy

A TDS test was carried out using the apparatus (HTDS-003). After electrochemical hydrogen permeation, the temperature of 0.055 wt% Nb-X80 steel samples rose to 700 °C at a rate of 100 °C/h; thus, the relationship curve between the desorption rate and temperature was obtained.

#### 2.2.3. Electrochemical Hydrogen Permeation

**Figure 1:**
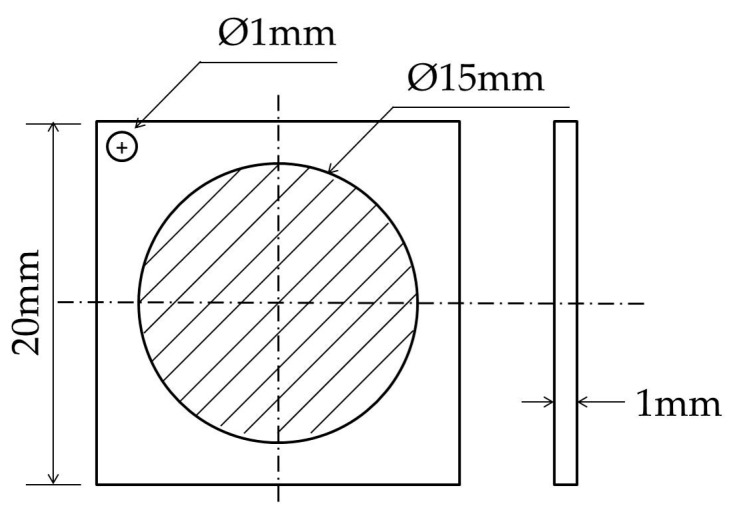
Dimension of specimens for electrochemical hydrogen permeation.

Both test steels were machined into 20 × 20 × 1 mm^3^ squares, and an R = 0.5 mm hole, to connect the wires, was machined at one of the apexes, as shown in Figure 1. Both sides of the samples were ground with sandpaper, polished, rinsed with deionized water, degreased ultrasonically for 10 min in acetone, and then rinsed again and dried. Finally, the samples were coated with nickel on one side using a Watt solution.

**Figure 2:**
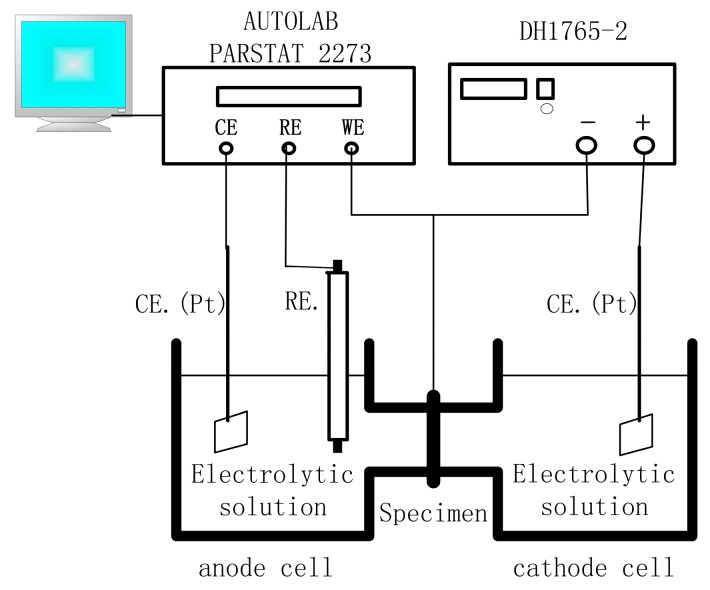
Electrochemical hydrogen permeation device.

A Devanathan-Stachurski double electrolytic cell was used in the experiment, as shown in Figure 2. It consisted of an anode cell and a cathode cell. The sample was fixed between two cells, and the nickel-plated sides faced the anode cell. The exposed test area was 1.76 cm^2^, as shown in Figure 2. The solution of the anode cell was 0.2 M NaOH, and the cathode cell was a mixed solution of 0.2 M NaOH and 0.25 g/L thiourea. Thiourea was used as a poisoning agent in order to prevent hydrogen atoms from entering the hydrogen molecules and escaping. The cathode cell was connected to a constant potential instrument (DH1765-2). The anode cell was connected to an electrochemical workstation (Autolab Parstat 2273). A saturated calomel electrode was selected as the reference electrode, and platinum electrode served as the counter electrode. 300 mV was applied to the anode surface. When the anode current density was less than 0.2 µA/cm^2^, a constant current density 2 mA/cm^2^ was applied to the cathode surface. Simultaneously, the I(t) versus t curve, namely the anode current against the hydrogen permeation time, was recorded.

In order to study the hindering effects of different hydrogen traps in Nb-bearing steel, three steps of hydrogen permeation were applied sequentially in the same sample. In the first step, the permeation was operated after the sample was heat-treated for 3 h at 600 °C. The second step was as follows: firstly, the permeated sample was heated to the critical temperature T_c_, which divides reversible and irreversible traps, and held for 2 to 3 h; then hydrogen permeation was operated. In the final step, the sample was put in an oven at 30 °C for about one day, and then subjected to the last permeation.

## 3. Results and Discussion

### 3.1. Microstructure and Precipitates

**Figure 3:**
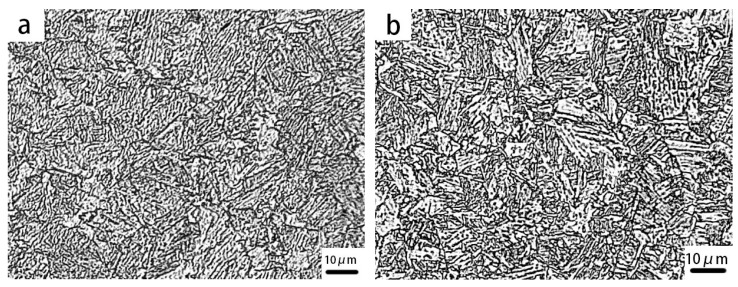
Microstructures of Nb-bearing X80 (**a**) and Nb-free X80 (**b**).

**Figure 4:**
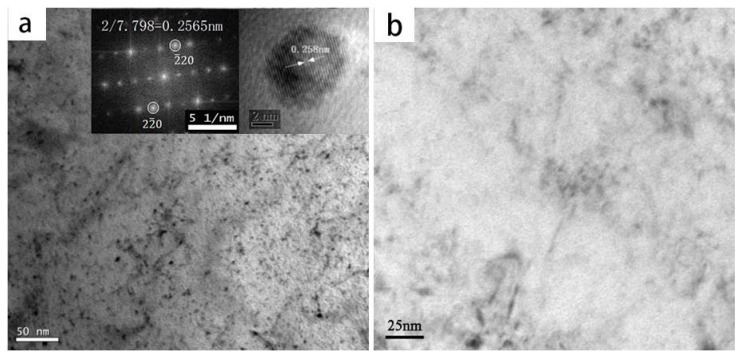
TEM images of nanosized precipitates in Nb-bearing X80 (**a**) and Nb-free X80 (**b**).

Both test steels possessed the same chemical compositions, except for Nb. As shown in Figure 3, the microstructures of both samples were granular bainite (GB), which consisted of a matrix of bainite ferrite (BF) and martensite/austenite island-liked phases. A slight dissimilarity existed in the specimens. The microstructure of Nb-bearing X80 was more homogeneous and finer than that of the Nb-free steel. Moreover, as shown in Figure 4a, a large number of precipitates, with sizes between 3 and 10 nm, were dispersed in the BF matrix of Nb-X80 steel. On the other hand, the matrix of the Nb-free steel hardly contained any precipitates, as shown in Figure 4b. The electron diffraction patterns and a high-resolution photograph of the precipitates, shown in Figure 4a, revealed that the precipitate was niobium carbide, which was coherent with the matrix. The results confirmed that NbC precipitates, fined down to several nanometers, can form coherent interfaces with the bcc-Fe matrix. On the other hand, the misfit of fcc-NbC and bcc-Fe, closing to the coherent interface, induced a stronger strain field than that of semi-coherent and incoherent interfaces. The coherent interface possessed the highest strain energy, but the lowest interfacial energy, and acted as the most effective irreversible trap for hydrogen. Semicoherent NbC demonstrated a weaker capacity for hydrogen trapping, and incoherent NbC was unable to trap hydrogen [15].

### 3.2. Hydrogen Diffusion in Steel with and without NbC

**Figure 5:**
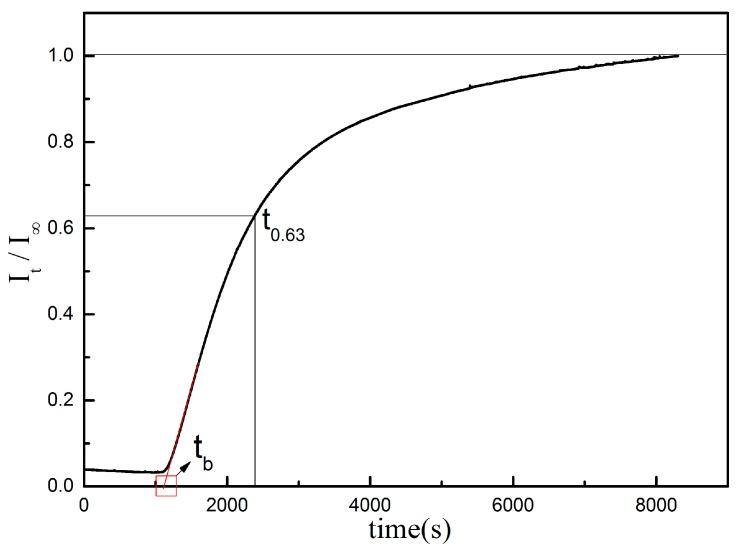
Schematic diagram of hydrogen permeation curve.

The diffusion of hydrogen inside the sample is consistent with Fick’s law,

(1) $$J=-D\frac{\partial C}{\partial x},\;\frac{\partial C}{\partial t}=D\frac{\partial^{2}C}{\partial x^{2}}$$

where J is the diffusion flux, J = i/F, i is the current density, F is the Faraday constant, and D is the diffusion coefficient. In addition, the boundary conditions of the hydrogen diffusion in the sample are:

$$C\left(x,t=0\right)=0,\;C\left(0,t\right)=C_{0},\;C\left(L,t\right)=0$$

where L is the specimen thickness. Using the solution, the apparent diffusion coefficient can be obtained:

(2) $$D=\frac{L^{2}}{6t_{0.63}}$$

(3) $$D=\frac{L^{2}}{15.3t_{b}}$$

where t_0.63_ is the time corresponding to $\frac{I_{t}}{I_{\infty }}=0.63$; t_b_ is the penetration time, which is the time of the intersection between the extension of the initial straight line and the abscissa, which can be used to determine the nature of the trap. Figure 5 shows the schematic diagram of hydrogen permeation curve.

The surface hydrogen concentration is:

(4) $$C_{0}=\frac{{\mathrm{Li}}_{\infty }}{\mathrm{DF}}$$

In this work, Equation (2) was used to calculate D_app_.

**Figure 6:**
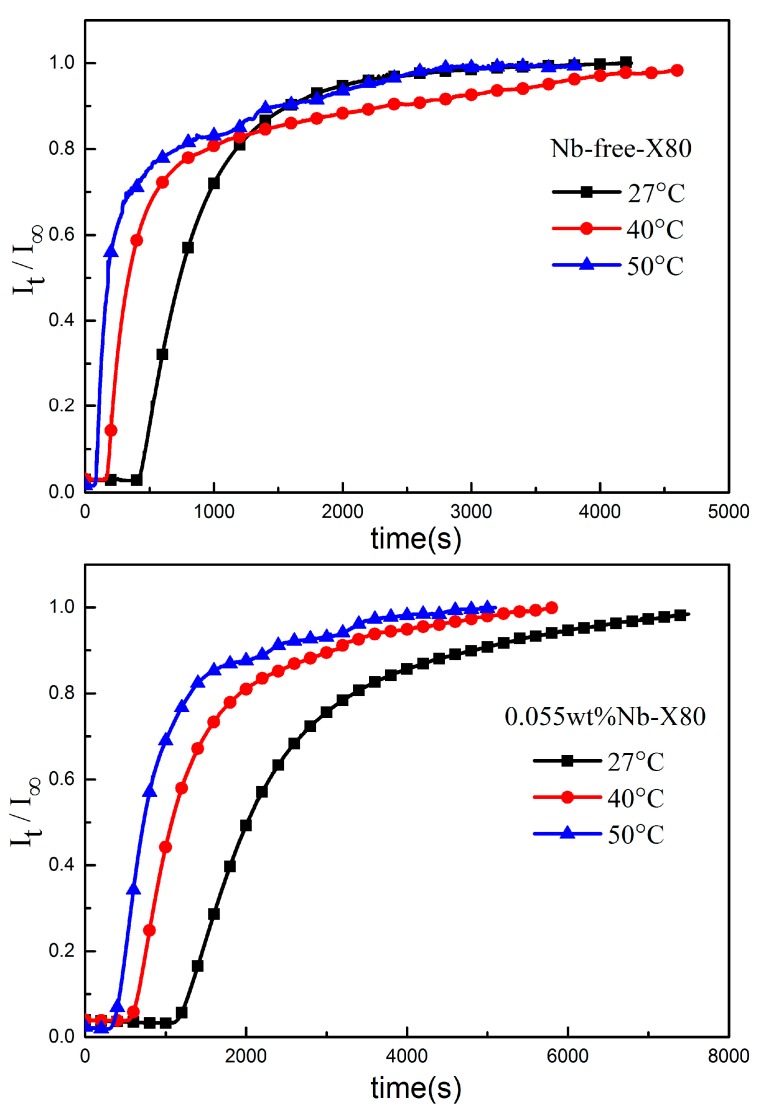
Hydrogen permeation curves of the samples at different temperatures.

The electrochemical hydrogen permeation of Nb-free X80 and Nb-bearing X80 steel samples were carried out at 27 °C, 40 °C, and 50 °C. The normalized curves of the anode current density versus the hydrogen permeation time are shown in Figure 6. Using Equations (2) and (4), the D_app_ of hydrogen and other parameters were obtained, as shown in Table 2.

From above data, it can be seen that, as temperature increased, steady-state current density I_∞_ increased, penetration time t_b_ and lag time t_0.63_ shortened, and hydrogen diffusion coefficient D_app_ was enhanced. Hydrogen atoms penetrated the sample more effectively because of the increase of kinetic energy as the temperature rose.

The diffusion theory shows that the relationship between diffusion coefficient D of the atom and diffusion temperature T is consistent with the Arrhenius equation:

(5) $$D=D_{0}e^{-\;\frac{Q}{\mathrm{RT}}}$$

According to this equation, the linear relationship between lnD and 1/T can be obtained [19].

(6) $$\mathrm{InD}={\mathrm{InD}}_{0}-\frac{Q}{\mathrm{RT}}$$

Based on fitting the data of Nb-free-X80 and 0.055 wt% Nb-X80 test steel in Table 2, the relationships between the apparent diffusion coefficient of hydrogen and the temperature were deduced, as follows.

For Nb-free-X80 test steel:

(7) $$\mathrm{lnD}=-\frac{5024.89}{T}+3.57{\;\mathrm{cm}}^{2}/s$$

For 0.055 wt% Nb-X80 test steel:

(8) $$\mathrm{lnD}=-\frac{4757.94}{T}+1.73{\;\mathrm{cm}}^{2}/s$$

Both fitting coefficients, Adjusted R-Square, were above 0.99.

Thus far, some different hydrogen diffusion coefficients in Nb-bearing pipeline steel have been reported: For example, 16 × 10^−6^ cm^2^/s in 0.047 wt% Nb low-alloy pipeline steel, and 9.24 × 10^−7^ cm^2^/s in 0.04 wt% Nb X65 steel [12,20]. In our work, the D_app_ of 0.055 wt% Nb-X80, with 3~10 nm precipitates, was 6.97 × 10^−7^ cm^2^/s at 27 °C. This difference may be caused by the composition of the matrix, and the size and quantity of NbC or NbN. Our results provide an alternative and targeted formula for hydrogen diffusion in high-strength low-alloy pipeline steels with the microalloying element Nb.

Moreover, the hydrogen permeation curves of both samples, in Figure 6, and the data, in Table 2, reveal that the t_b_ and t_0.63_ of hydrogen diffusion in Nb-bearing X80 were much longer than those of Nb-free X80. The surface hydrogen concentration, determined using Equation (4), also showed a large difference in the steels.

The Nb-bearing X80 sample contained a large number of NbC precipitates, which acted as irreversible traps, and there were more grain boundaries than Nb-free X80, so that the hydrogen diffusion was hindered markedly. The reason for this was that hydrogen atoms were firstly captured by the deep traps in the strain field around the interface of the NbC precipitates and the matrix, and then by reversible traps, such as grain boundaries, dislocations, and so on, finally, hydrogen saturated in the traps and diffused through the crystal lattice. As a result, penetration time t_b_ and lag time t_0.63_ turned out to be relatively longer in 0.055 wt% Nb-X80 in the hydrogen permeation curve, so that the D_app_ of hydrogen in Nb-bearing X80 was much lower at each temperature. Because of these irreversible traps and greater numbers of grain boundaries, it can be seen in Figure 6 that the hydrogen in 0.055 wt% Nb-X80 steel reached a stable value more slowly than in Nb-free steel.

### 3.3. Effects of Traps on Hydrogen Diffusion

#### 3.3.1. Types of Hydrogen Trap

The addition of Nb increased irreversible hydrogen traps in steel, but the temperature boundary between the reversible traps and the irreversible traps of 0.055 wt% Nb-X80 steel was unclear. The binding energy was related to temperature, so the temperature could be directly classified using the TDS method. In the TDS curve, the peak at lower temperatures formed due to hydrogen desorption in the reversible traps, while hydrogen desorption in the irreversible traps, induced by NbC precipitates, caused the peak at higher temperatures [14].

**Figure 7:**
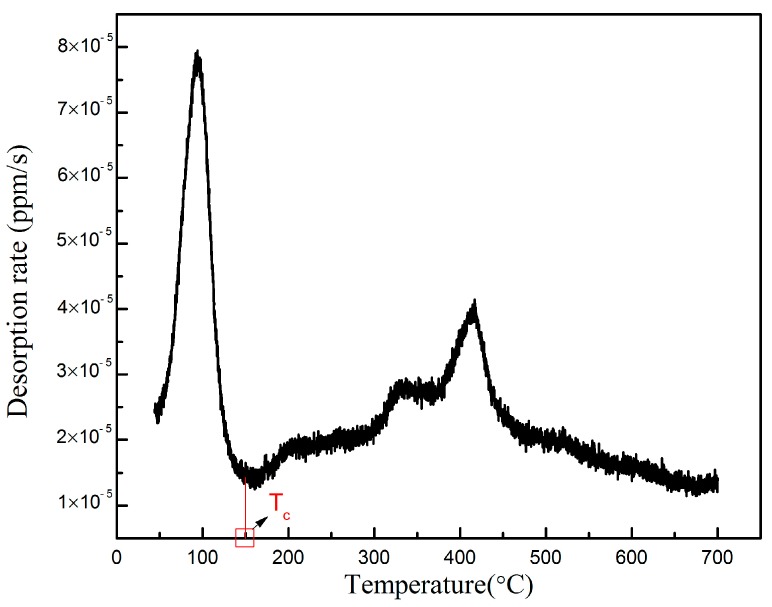
TDS curve of 0.055 wt% Nb-X80 sample.

Figure 7 shows the TDS curve of the hydrogen desorption rate versus temperature of 0.055 wt% Nb-X80 steel after electrochemical hydrogen permeation. Therefore, according to the desorption curve of hydrogen, 150 °C was considered to be the critical temperature (T_c_) to divide reversible traps and irreversible traps: Hydrogen that escaped before 150 °C was considered to be in the reversible traps, such as grain boundaries; and hydrogen that escaped over 150 °C was thought to be in the irreversible traps. Due to the constant heating rate, the integral of the TDS curve can represent the amount of hydrogen desorption. The integral of the TDS curve from 50 to 150 °C represents hydrogen fixed in the reversible traps, while, that of over 150 °C represents hydrogen fixed in the irreversible traps. Origin9.0 was used to perform the integral operation. The ratio of hydrogen in the reversible traps and irreversible traps was determined to be 4:9.

#### 3.3.2. Hindering Effects of Traps on Hydrogen Permeation

For further study of the effects of NbC as an irreversible trap on hydrogen diffusion, different hydrogen trap types in Nb-bearing steel were designed using different heat treatments [21,22]. According to the TDS curve and T_c_, three steps of hydrogen permeations were applied as mentioned in Section 2.2.3. In the first step, hydrogen atoms remaining in the metallurgical process in sample were driven out at 600 °C, all of the irreversible and reversible traps, such as NbC, grain boundary, and others, absorbed hydrogen in the following permeation. In the second step, when the sample was held at 150 °C, hydrogen atoms in reversible traps were driven out, and those in the irreversible traps were preserved. Only reversible traps and the lattice needed to be permeated with hydrogen in the following process. In the third step, after the diffusible hydrogen in the lattice escaped at 30 °C, hydrogen atoms only diffused in very shallow traps and lattices when the sample was permeated.

**Figure 8:**
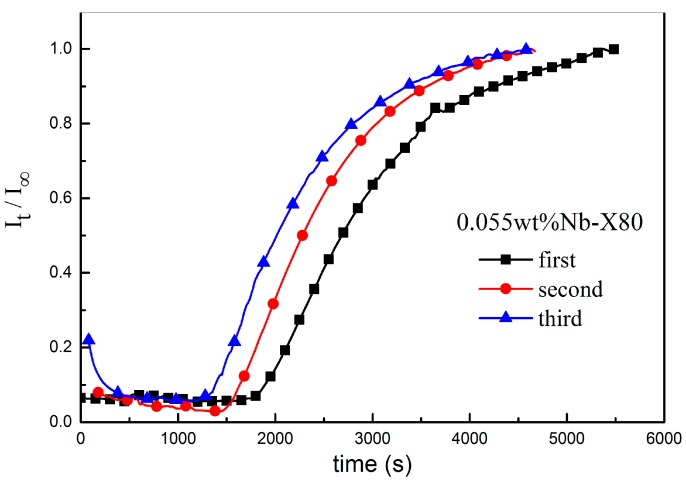
Anode normalization curves of 0.055 wt% Nb steel in different steps.

The electrochemical hydrogen permeation on 0.055 wt% Nb steel was conducted in the above mentioned three steps at room temperature (~23 °C). The apparent diffusion coefficient of hydrogen and the surface hydrogen concentration in the sample were calculated using Foucault’s law. As shown in Figure 8, three normalized curves were obtained. The diffusion characteristic parameters calculated using Fick’s law are shown in Table 3.

It can be seen from Figure 8 and Table 3, t_b_ and t_0.63_ in the first permeation were the longest, and the corresponding D_app_ of hydrogen was the smallest. The D_app_ of hydrogen shows a significant difference in all three steps. Comparing the results in different types of hydrogen traps, it can be found that NbC precipitates as irreversible hydrogen traps caused the difference between the first and the second permeations; reversible hydrogen traps, such as grain boundary and dislocation, caused the difference between the second and the third permeations. The results confirmed the marked hindering effect of deep traps formed by nanosized and coherent NbC precipitates on hydrogen diffusion in Nb-bearing X80 pipeline steel. The reversible traps obviously influenced hydrogen diffusion in steel at room temperature.

## 4. Conclusions

1. The relationship between the diffusion coefficient of hydrogen and temperature in Nb-free X80 steel was established as lnD = −5024.89/T + 3.57 cm^2^/s, and the relationship in 0.055 wt% Nb X80 steel was found to be lnD = −4757.94/T + 1.73 cm^2^/s. The equation of Nb-bearing steel provides an alternative for the prediction of hydrogen diffusion in high-strength low-alloy steels with the Nb microalloying element.
2. For 0.055 wt% Nb X80 steel, the critical temperature between the reversible and irreversible traps was measured as 150 °C, and the ratio of hydrogen captured by the reversible traps, relative to that of the irreversible traps, was determined to be 4:9.
3. The irreversible hydrogen traps in Nb-bearing X80 steel were formed by NbC precipitates with sizes of 3~10 nm, which were misfit and were coherent with the bcc-Fe matrix. The irreversible traps markedly hindered hydrogen diffusion in steel.

## Figures and Tables

**Table 1:** Chemical composition of test steels (mass fraction).

Material	C	Si	Mn	P	S	Nb	Ti	Mo	Ni	Cu
Nb-free	0.058	0.28	1.85	0.004	0.006	0	0.016	0.26	0.26	0.26
0.055 wt% Nb	0.060	0.27	1.84	0.004	0.005	0.055	0.015	0.25	0.26	0.26

**Table 2:** Apparent diffusion coefficient and the surface hydrogen concentration of two samples at different temperatures.

Parameters		T/°C		
		27	40	50
Nb-free-X80	Penetration time t_b_/s	403	156	80
	Lag time t_0.63_/s	869	444	263
	Apparent diffusion coefficient D_app_/(cm^2^·s^−1^)	1.92 × 10^−6^	3.75 × 10^−6^	6.34 × 10^−6^
	Stable current density I_∞_/(µA·cm^−2^)	4.88	6.39	8.32
	Surface hydrogen concentration C_0_/(µmol·cm^−3^)	2.63	1.77	1.36
0.055 wt% Nb-X80	Penetration time t_b_/s	1135	565	370
	Lag time t_0.63_/s	2390	1301	883
	Apparent diffusion coefficient D_app_/(cm^2^·s^−1^)	6.97 × 10^−7^	1.28 × 10^−6^	1.89 × 10^−6^
	Stable current density I_∞_/(µA·cm^−2^)	4.99	7.31	7.96
	Surface hydrogen concentration C_0_/(µmol·cm^−3^)	7.42	5.92	4.37

**Table 3:** Diffusion characteristic parameters obtained by hydrogen permeation of 0.055 wt% Nb steel in different steps.

Parameters	First	Second	Third
penetration time t_b_/s	1698	1468	1236
lag time t_0.63_/s	2990	2542	2282
D_app_/(cm^2^ s^−1^)	5.57 × 10^−7^	6.56 × 10^−7^	7.30 × 10^−7^
